# Self-Management Combined With Digital Health Interventions to Improve Dietary Behavior, Exercise Behavior, Stress Management Behavior, and Blood Pressure Among Thais With Uncontrolled Hypertension: Protocol for an Explanatory Sequential Study

**DOI:** 10.2196/81148

**Published:** 2026-01-02

**Authors:** Saowaluck Sukpattanasrikul, Naruemol Singha-Dong, Yajai Sitthimongkol

**Affiliations:** 1 Department of Fundamental Nursing, Faculty of Nursing, Mahidol University, Nakhon Pathom, Thailand; 2 School of Community Health Nursing, Institute of Nursing, Suranaree University of Technology, 111 University Avenue, Suranari subdistrict, Muang, Nakhon Ratchasima, 30000, Thailand, 66 4422 3514, 66 4422 3506; 3 Department of Mental Health and Psychiatric Nursing, Faculty of Nursing, Mahidol University, Nakhon Pathom, Thailand

**Keywords:** uncontrolled hypertension, self-management, digital health intervention, web-based application, dietary behavior, exercise behavior, stress management behavior, blood pressure, explanatory sequential design

## Abstract

**Background:**

Uncontrolled hypertension causes substantial morbidity and mortality as well as rising health care costs. Adherence to self-management is critical for minimizing the risk of hypertensive complications. Traditional self-management is hindered by delayed management and insufficient support. Digital health interventions offer a feasible solution for closing these gaps and enhancing hypertension self-management. Little is known about patients’ perspectives and experiences concerning how digital health interventions influence their self-management.

**Objective:**

This study aims to determine the effectiveness of a self-management intervention combined with digital health interventions on dietary behavior, exercise behavior, stress management behavior, and blood pressure among Thais with uncontrolled hypertension. The study explored and compared experiences and perceptions among participants with varying levels of blood pressure control.

**Methods:**

This study uses an explanatory sequential design performed in 2 phases comprising (1) a quasi-experimental design with 2 groups using repeated measures to determine the effects of self-management combined with digital health intervention and (2) an in-depth interview approach to explore the perceptions and experiences of 24 participants regarding the combination of self-management and digital health interventions after the intervention. In phase 1, participants were allocated by lottery to either the intervention group, which underwent an 8-week self-management intervention combined with digital health interventions, or the control group. The Dietary Approaches to Stop Hypertension Questionnaire, the Exercise Behavior Questionnaire, the Brief COPE inventory (Thai Version), and an automatic blood pressure measurement were used for data collection at baseline and the 4th and 8th weeks. In phase 2, semistructured interviews were used to conduct in-depth interviews. The analysis will take into account the effects of the interventions on dietary, exercise, and stress management behaviors, as well as blood pressure, using generalized estimating equations and linear mixed-effects modeling. We will perform the method by Colaizzi for the qualitative portion of the analysis.

**Results:**

Funded in December 2024, this study recruited 86 patients with uncontrolled hypertension at the Siriraj Primary Care Unit, Thailand. This study received ethical approval on May 31, 2025, and participant recruitment began in August 2025. In phase 1, this study began recruiting participants in August 2025, with data collection occurring from August through the first half of November 2025. Phase 2 was completed at the end of November 2025. The data analysis is expected to be completed by December 2025. The expected date for the results to be submitted for publication is March 2026.

**Conclusions:**

This study has the potential to address the gap between traditional self-management and digital health interventions for improving self-management behaviors and reducing blood pressure. The findings may offer practical guidance for nurses and other health care providers for managing uncontrolled hypertension in Thailand and contribute valuable insights for shaping future health care policies.

## Introduction

Uncontrolled hypertension (blood pressure [BP]≥140/90 mm Hg) is a substantial health problem that presents a challenge for the global health system, including in Thailand. Approximately 26% of patients with hypertension worldwide have uncontrolled hypertension [1]. Thailand follows this trend, with 24.6% of the population with hypertension having uncontrolled hypertension, often linked to lifestyle-related factors [1,2]. In Thailand, 15% of Thais with uncontrolled hypertension experience complications such as cardiovascular disease, stroke, or chronic kidney disease [3]. Moreover, hypertension is associated with an average cost of ฿964 (US $30.96) per visit, resulting in a total expenditure of ฿1.4367 billion (US $46,135,167) [4]. In Thailand, like most other countries, uncontrolled hypertension is associated with poor self-management, such as an unhealthy diet, a lack of exercise, and inappropriate stress management [5-7]. A study by Sodkhomkham [8] found that self-management, including dietary behavior, exercise behavior, stress management, and medication adherence, could predict BP control with an accuracy of 82%. Thus, focusing on self-management is indispensable for addressing uncontrolled hypertension.

Self-management is widely recognized as a pivotal aspect of improving self-management behaviors and clinical outcomes in hypertensive care [9]. Successful hypertension self-management requires interactive group education on hypertension and its management, which can be linked to the condition, monitoring with feedback, the provision of equipment, and lifestyle advice with support related to diet and exercise, weight reduction, stress management, and medication adherence [9]. Nurses play a crucial role in supporting patients with uncontrolled hypertension by providing health education, counseling, coaching, and facilitation of support [10-12]. However, a study by Liang et al [13] illustrated that traditional hypertension self-management is less effective due to the barriers to communication between health care providers and patients, a lack of timely and detailed management, and a lack of instrumental support. Likewise, time constraints and heavy workloads hinder nurses’ ability to prioritize hypertension management while providing patient care [14]. To overcome these limitations, digital health interventions (DHIs) serve as a complementary method to support self-management [15].

DHIs have emerged as effective, beneficial, and widely accessible strategies for supporting hypertension self-management [16,17] and promoting health equity for patients with uncontrolled hypertension [17]. The World Health Organization (WHO) [18] emphasized the importance of patient-centered DHIs, including interactive voice response systems, phone calls, web-based telecare platforms, smartphone apps, SMS, and multimedia messaging services. Nurses play a pivotal role in promoting self-management to prevent and control hypertension. They empower individuals to adopt and maintain healthy behaviors through comprehensive education, personalized goal setting, and ongoing support [19]. Nurses meticulously assess risk factors, provide tailored counseling, and leverage digital health tools to help patients effectively monitor their conditions [20]. By consistently following up with patients and coordinating care, nurses enhance patient engagement, adherence, and, ultimately, long-term health outcomes [20]. An updated systematic review by Sukpattanasrikul et al [21] highlighted that the most effective DHIs for patients with uncontrolled hypertension target multiple health behaviors, incorporating key components such as health education, reminders, self-monitoring, feedback, and instrumental support.

Investigation into previous self-management combined with DHIs used for patients with uncontrolled hypertension between 2014 and 2024 revealed that most previous studies conducted research in an effort to control hypertension through self-management education, including health education related to the disease and self-care management [22-24]; self-management skill development through goal setting, decision-making, action plans, self-monitoring, and self-evaluation [23,25,26]; and providing instrumental support such as automatic BP measurement [27], salt meters [28], and graphics displaying BP over time [29]. Concurrently, DHIs being used to support self-management include health education [22,30], medication reminders [22,30-33], and BP monitoring reminders [27,31,33,34], as well as consultation through social media platforms such as WeChat [35] and Line applications [32].

This study identified a gap in knowledge among Thai patients with uncontrolled hypertension concerning self-management combined with DHIs by reviewing the literature over 1 decade (2014‐2024). Traditional self-management approaches within service units have limitations due to their group-based nature. They may not adequately address the specific challenges and requirements of patients with uncontrolled hypertension. Additionally, providing consultation through the Line application increases the workload for health care personnel, who must manually review information from patients with hypertension and respond [19]. Integrating automated response programs (chatbots) into consultations can help patients with uncontrolled hypertension seek advice or consultations tailored specifically to their needs and receive it more quickly [19].

Literature reviews have also shown that providing health education through applications alone increases patients’ knowledge of hypertension [36] but fails to control BP levels within the recommended range (BP<140/90 mm Hg) [37]. Meanwhile, the perceptions and experiences of patients regarding the use of DHIs to support self-management processes remain unclear. Thus, this study addresses the knowledge gap by implementing multiple interventions that integrate self-management with DHIs for patients with uncontrolled hypertension, emphasizing a hybrid reinforcement model. This approach has the potential to offer nurses and other health care providers practical guidance on how to care for patients with uncontrolled hypertension, thereby improving their hypertensive outcomes. This study could also provide important information leading to the further development of health care policies in Thailand.

The objectives of this study are (1) to determine the dietary, exercise, and stress management behaviors, as well as BP, among Thais with uncontrolled hypertension after receiving a self-management intervention combined with DHIs and (2) to explore their experiences and perceptions after receiving the interventions. More specifically, this study aims to (1) evaluate the effectiveness of self-management combined with DHIs for improving dietary, exercise, and stress management behaviors, as well as BP, among Thais with uncontrolled hypertension and (2) explore the perceptions and experiences of study participants regarding the combined self-management and DHIs after the intervention and to compare these perceptions and experiences with varying levels of BP control.

## Methods

### Overview

The study uses an explanatory sequential design to determine the effectiveness of self-management combined with DHIs among patients with uncontrolled hypertension, exploring the experiences and perceptions after receiving the intervention among patients with uncontrolled hypertension who received treatment at Siriraj Primary Care Unit. The study achieves its objectives in 2 phases: phase 1, a quasi-experimental design with 2 groups and repeated measures, and phase 2, an in-depth interview approach.

**Figure 1.**
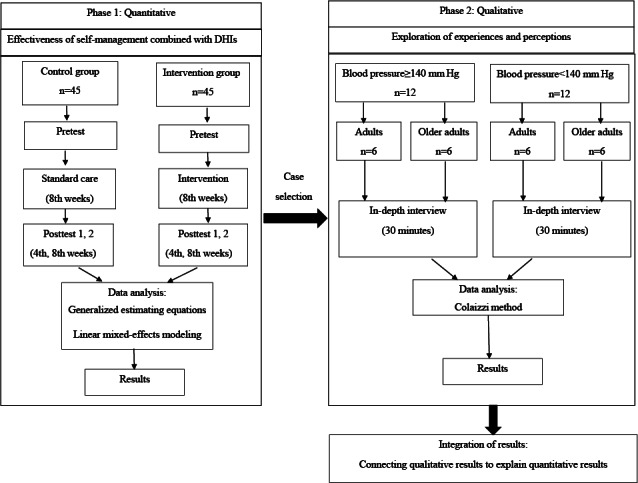
Explanatory sequential design. DHI: digital health intervention.

The integration of the 2 phases aligns with an explanatory sequential design. First, the connecting integration was applied to the sampling design. The researcher determined the participants in phase 2 by purposively selecting them based on phase 1 results (eg, BP≥140/90 mm Hg or BP<140/90 mm Hg) and age group, to ensure we capture a range of perceptions and experiences. Second, after both phases are completed, the integration of results will take place. As to the purpose of this protocol, the qualitative themes are used to explain dietary, exercise, and stress management behaviors, as well as BP, among Thais with uncontrolled hypertension. Joint displays and narratives will integrate quantitative and qualitative information, integrating outcome patterns with significant themes to draw conclusions on how self-management and DHIs impact outcomes (Figure 1).

### Phase 1: Quantitative Method to Evaluate the Effectiveness of Self-Management Combined With DHIs

This study involves a quasi-experimental design comparing 2 groups with repeated measures to determine the effectiveness of an 8-week self-management program combined with DHIs on dietary behavior, exercise behavior, stress management behavior, and BP.

#### Recruitment

Participant recruitment included poster advertisements at the Siriraj Primary Care Unit, plus an announcement of the invitation at the Siriraj Primary Care Unit. Patients with uncontrolled hypertension who were interested in participating in the project could express their interest to the nurse at the Siriraj Primary Care Unit or the researchers. The researchers then screened patients with hypertension according to the inclusion and exclusion criteria. The study approached those who passed all inclusion criteria individually to sign an informed consent document. Participants who met the inclusion and exclusion criteria were allocated to either the control or experimental group. The recruitment protocol enphasized that participants could opt out at any time before, during, or after participation, without consequences from the Siriraj Primary Care Unit. In addition, participants who attended fewer than 75% of the activities (less than 6 weeks out of 8 weeks) were withdrawn from this study without consequences from the Siriraj Primary Care Unit.

#### Participants

The study selected participants by proportional stratified random sampling, followed by purposive sampling adhering to the inclusion criteria. The inclusion criteria were ages 45 years to 79 years old, previous history of a diagnosis of mild to moderate hypertension for at least 1 year and recorded in the database at the Siriraj Primary Care Unit, a determination of uncontrolled hypertension according to the BP criteria (BP 140/90 mm Hg to 179/100 mm Hg) for 2 consecutive measurements among patients with hypertension who received pharmacological and nonpharmacological treatments at the Siriraj Primary Care Unit, had an intention to change self-management behaviors, could be accessed by smartphone, and ability to communicate in Thai. The study excluded those with any severe complications such as cardiovascular, cerebrovascular, and end-stage renal disease; inadequate digital health literacy; cognitive impairment; moderate to total dependency for undertaking basic activities of daily living; an inability to use a telephone or manage their medications; and similar characteristics but were previously exposed to education programs. Additionally, the study excluded participants who experienced abnormal symptoms during the activity, such as dizziness or fainting, and who did not improve after receiving first aid. The study withdrew participants who attended the activities for fewer than 75% of the time (less than 6 weeks out of 8 weeks). Regarding participant allocation, this study used the lottery method to determine the days to assign research participants to either the intervention or control group. The study designated the first and second lottery draws as the control group (Monday and Thursday), with the third and fourth lottery draws designated as the intervention group (Tuesday and Wednesday).

This study requires a total of 90 patients with uncontrolled hypertension to achieve a representative sample for phase 1, and we used the G*power program version 3.1.9.7 [38] to determine the estimated sample size based on repeated-measures ANOVA. An effect size (*f*) of 0.25 was set [39], with a significance level of .05, power of .80, the number of groups set at 2, the number of measurements set at 3, and the correlation among repeated measures set at 0.5. Therefore, the determined total sample size was 86 participants, with 43 participants per group. A previous study showed that patients with uncontrolled hypertension dropped out of the self-management program at a rate of 5% [39]. Finally, the total sample size was determined to be 90. Next, the proportional stratification was calculated using the following formula: sample size = (sample size/population size) × stratum size. Thus, in this study, 18 adults with uncontrolled hypertension and 72 older adults with uncontrolled hypertension were selected as the sample.

#### Research Measurements

The study used research instruments consisting of (1) screening measurements, (2) experimental measurements, and (3) outcome measurements. The details are presented in the following sections.

##### Screening Measurements

###### Cardiovascular Prevention Stage of Change Questionnaire

The study used the cardiovascular prevention stage of change questionnaire to screen the readiness of patients with uncontrolled hypertension to change their behavior, with permission from the developers. Singha-Dong et al [40] developed this measurement. It consists of 13 items, with response options reflecting the stages of behavior change, ranging from 0 points (not considering change) to 5 points (maintaining the behavior for more than 6 months). The total score ranges from 0 to 65. Regarding its psychometric properties, the test-retest reliability is 0.91.

###### Thai Version of the Mini-Cog

The study used the Thai version of Mini-Cog for screening for cognitive impairment of Thais with uncontrolled hypertension, with permission from the developers and translators. Borson et al [41] developed this measurement, and Trongsakul et al [42] subsequently translated it into the Thai version. It consists of 2 sections, including a memory test (3-item word recall) and a clock-drawing test. The memory test is assigned a score of 1 point per item, giving it a total of 3 possible points. The clock-drawing test is assigned a score of 2 points, giving it a total of 2 possible points. The scores are calculated from these 2 sections. Therefore, the total possible score for the Thai version of the Mini-Cog is 5 points. Possible scores range from 0 to 5, with a cutoff point of ≥3 indicating cognitive intactness. Regarding its psychometric properties, the Mini-Cog has shown high sensitivity (99%) and specificity (96%) [41].

###### eHealth Literacy Scale (eHEALS)

The study used the eHealth Literacy Scale (eHEALS) to screen the digital health literacy of patients with uncontrolled hypertension, with permission from the developers. Norman and Skinner [43] developed this measurement, and the Office of the Department of Health [44] subsequently translated it into the Thai version. It consists of 8 items rated on a Likert scale from 1 (strongly disagree) to 5 (strongly agree). Possible scores range from 8 to 40, with a cutoff point of ≥26 indicating adequate digital health literacy [45]. Regarding its psychometric properties, the test-retest reliability is 0.85 [46].

Participants must score 26 or higher on the eHEALS to demonstrate their intention to utilize digital tools throughout the screening process. This threshold ensures that participants can safely and effectively engage with the digital intervention. In practice, this threshold is used to maximize participation and minimize dropout rates during the intervention phase. This is crucial for the initial assessment of the intervention’s effectiveness.

###### Thai Version of the Barthel Index

The study used the Barthel index-Thai version to screen activities of daily living of participants who are aged 60 years through 79 years, with permission from the developers and translators. Mahoney and Barthel [47] developed this instrument, and Jitapankul and colleagues [48] subsequently translated it into the Thai version. It consists of 10 items, including feeding, grooming, transfer, toilet use, mobility, dressing, stairs, bathing, bowels, and bladder. The minimum score for the Barthel index is 0, and the maximum score is 20. The Barthel index categorizes dependency as totally dependent (0 to 4 points), severely dependent (5 to 8 points), moderately dependent (9 to 11 points), mildly dependent (12 points or more), and independent (20 points). Regarding psychometric property, the Kappa coefficients for the interrater reliability test were 0.79 and 0.68, respectively [48].

###### Lawton-Instrumental Activities of Daily Living Scale-Thai Version

The study used the Lawton-Instrumental Activities of Daily Living Scale-Thai version to screen independent living skills for patients who were aged 60 years to 79 years, with permission from the developers and translator. Lawton and Brody [49] developed this instrument, and Phanasathit [50] subsequently translated it into the Thai version. It consists of 8 items: using a telephone, shopping, food preparation, housekeeping, laundry, transportation, responsibility for own medications, and handling finances. The summary score ranges from 0 (low function, dependent) to 8 (high function, independent). Regarding the psychometric property, the Kappa coefficient of interrater reliability test was 0.85, with a Cronbach α reliability of 0.32 (95% CI –0.12 to 0.66), and the test-retest reliability according to the Spearman rank correlation coefficient was 0.46 [50].

### Experimental Measurements

The study established a self-management intervention combined with DHIs, including a web-based application, chatbot, salt meter, and smartwatch. The study developed self-management skills combined with DHIs according to the Integrated Theory of Health Behavior Change [51]. The feasibility of this program for objective congruence was examined by 3 experts, including a nurse instructor specializing in self-management, a nurse instructor specialist experienced in hypertensive care, and an instructor specializing in digital health. The index of congruence was 1.00. A team of software developers developed the web-based application and chatbot. Table 1 shows the details of the self-management intervention combined with the activities in the DHIs.

**Table 1.** Details of self-management combined with the digital health intervention activities.

Week	Activities
Week 1	Group activity, 5‐6 participants, 60 min Assessing the knowledge and health beliefs and then providing accurate information (15 min) Self-management skills development (20 min) Accessibility and utilizing support instruments, including a salt meter, a smartwatch, web-based application, and chatbot (25 min)
Weeks 2‐3	The chatbot follow-ups encourage self-care twice a week (Wednesday and Friday at 8 AM), offering personalized feedback and allowing participants to request one-on-one consultations with the researcher.
Week 4	Group activity, 5‐6 participants, 60 min Follow-up and review of self-management skills (20 min) Training about reading nutrition labels (20 min) Reviewing the web-based application and its functions, followed by a question-and-answer session (20 min)
Weeks 5, 6, and 7	The chatbot follow-ups encourage self-care twice a week (Wednesday and Friday, 8 AM), offering personalized feedback and allowing participants to request one-on-one consultations with the researcher.
Week 8	Group activity, 5‐6 participants, 60 min Knowledge sharing and self-management evaluation with goal comparison, followed by a question-and-answer session (30 min) Reviewing self-management skills (20 min) Encouragement to practice self-care at home (10 min)

### Outcome Measurements

#### Overview

At each time point in phase 1, the study collected outcome measurements including (1) the demographic data form (baseline), (2) Dietary Approaches to Stop Hypertension Questionnaire (DASHQ; baseline and 4th and 8th weeks), (3) Exercise Behavior Questionnaire (EBQ; baseline and 4th and 8th weeks), (4) the Brief COPE inventory (Thai version; baseline and 4th and 8th weeks), and (5) an automatic BP measurement (baseline and 4th and 8th weeks).

#### Demographic Data Form

The researchers developed the demographic data form to collect the participants’ demographic data. This semistructured interview form includes questions regarding gender, age, marital status, education, religion, occupation, income, sources of income, adequacy of income, living arrangement, duration of hypertension, received medicines, and underlying diseases.

#### Dietary Approaches to Stop Hypertension Questionnaire

The study used the DASHQ to examine the dietary behaviors of the participants, with permission from the developers, Siriboonyarit et al [52]. This measurement consists of 13 items: 8 items for the positive approach and 5 for the negative approach. This measurement utilizes a Likert scale with 5 categories, from 1 to 5 (1=“not at all” and 5=“always”), for items related to the positive approach. The response choices for all items are standardized on a scale from 13 to 65, with a score from 13 to 30.33 reflecting low dietary behavior, a score from 30.34 to 47.67 reflecting moderate dietary behavior, and a score from 47.68 to 65 reflecting high dietary behavior. The content validity was 0.91, with a reliability Cronbach α coefficient of 0.80. This study examined the reliability of the measurement using 30 patients with uncontrolled hypertension and determined it using the Cronbach α coefficient before collecting the data.

#### Exercise Behavior Questionnaire

The study used the EBQ to examine the exercise behaviors of the participants, with permission from the developers, Pieasakran et al [53]. The EBQ consists of 10 items. The measurement utilizes a Likert scale of 5 categories, from 0 to 4 (0=“not at all” and 4=“always”) for items related to the positive approach. The response choices for all items are standardized on a scale from 0 to 40, with higher scores indicating better exercise behavior. The content validity is 0.80, with a Cronbach α coefficient reliability at 0.87. This study examined the measurement reliability using 30 patients with uncontrolled hypertension and determined it using the Cronbach α coefficient before collecting the data.

#### Brief COPE inventory (Thai Version)

The study used the Brief COPE inventory (Thai version) to document the stress management behaviors of the participants, with permission from the developers, Numsang and Tantrarungroj [54]. This measurement consists of 28 items, including active coping, planning, positive reframing, acceptance, humor, religion, using emotional support, using instrumental support, self-distraction, denial, venting, substance use, behavioral disengagement, and self-blame. This measurement uses a Likert scale of 4 categories, from 1 to 4 (1=“not at all” and 4=“always”). The response choices for all items are standardized on a scale from 28 to 112, with higher scores indicating better stress management behavior. Regarding the psychometric properties, the test-retest reliability was 0.70. This study examined the reliability of the measurement using 30 patients with uncontrolled hypertension and determined it using the Cronbach α coefficient before collecting data.

#### Automatic BP Measurement

This study took an automatic BP measurement using an HEM-9200T (Omron Healthcare Inc), along with the standard method for measuring BP based on the guidelines from the Thai Hypertension Society [55]. The standard calibrator lab at the clinical instrument testing center calibrated the automatic BP measurement for accuracy at the start of the intervention and during the study.

### Preparation of the Research Assistant

The research assistant was a nurse who was trained in data collection. Initially, the researcher explained the objectives and methods of the study, including human rights protection of the sample. Second, the research assistant received a questionnaire and instructions for collecting the data. Third, the research assistant was, if not already, trained in BP measurement following the Thai Hypertension Society guidance [55]. After training, the research assistant carried out data collection under real situations to demonstrate an understanding of the instructions.

### Data Collection

In phase 1, we used a quasi-experimental design comparing 2 groups with repeated measures to determine the effects of self-management combined with DHIs on dietary, exercise, and stress management behaviors and BP.

First, we asked 45 participants in the control group to complete their demographic data, dietary behavior, exercise behavior, stress-management behavior, and BP measurement as a pretest. They then received standard medical treatments in accordance with the standards of the Siriraj Primary Care Unit. After completion of the intervention (4th week) and a follow-up period (8th week), they completed post-test I and post-test II. The research assistant collected the data.

The study asked the 45 participants in the intervention group to provide their demographic data, as well as dietary behavior, exercise behavior, stress-management behavior, and BP measurement as a pretest. They then received 8 weeks of self-management combined with DHIs. After completion of the intervention (4th week) and a follow-up period (8th week), they completed posttest I and posttest II. The research assistant collected the data.

### Data Analysis

In phase 1, the analysis will include both the complete case and intention-to-treat analyses. A statistical software program will be used to analyze the data from this phase. The study uses descriptive statistics (eg, frequency, percentage, mean, and SD) to describe the demographic data and characteristics of the sample. The study will use the *χ*^2^ test or independent *t* test to compare differences in the demographic data and outcome variables between the control and intervention groups at baseline. If differences are identified in the demographic data, the study will set different variables as covariates to control for the confounding factors. This study will use generalized estimating equations to test the effects of self-management combined with DHIs on dietary, exercise, and stress management behaviors, as well as BP, compared between the control and intervention groups. This study will use linear mixed-effects modeling to test the effects of self-management combined with DHIs on dietary, exercise, and stress management behaviors, as well as BP, within the intervention group at baseline, after completion of the intervention, and after completion of a follow-up period.

### Phase 2: Qualitative Method to Explore Experiences and Perceptions After Receiving the Interventions

This study conducted in-depth interviews to collect the participants’ perceptions and experiences after completing all self-management activities combined with DHIs.

#### Recruitment

In Phase 2, the research assistant screened potential participants for eligibility based on the inclusion criteria and informed them of the results by telephone. If the potential participants were deemed eligible and willing to participate in the study, the researcher scheduled a meeting. The researcher then screened the qualifications of patients with hypertension according to the inclusion criteria. The study approached those who passed all inclusion criteria individually to sign the informed consent document.

#### Participants

The study recruited participants through purposive sampling using the inclusion criteria. The inclusion criteria for this phase included individuals aged 45 years through 79 years old who had a BP lower than 140/90 mm Hg after participating in self-management combined with DHIs or individuals aged 45 years through 79 years old who had a BP higher than 140/90 mm Hg after participating in self-management combined with DHIs. The study withdrew participants who experienced abnormal symptoms during the activity, such as dizziness or fainting, that did not resolve after receiving first aid.

Additionally, the study determined the sample size for phase 2 in accordance with the data collection procedure. The minimum sample size for an in-depth interview approach is 12 participants, which aligns with the qualitative research principle that emphasizes data saturation [56]. However, the objective in phase 2 is to explore participants’ perceptions and experiences after receiving self-management combined with DHIs, stratified by age group and BP status. The researcher used a stratified approach, interviewing 6 adults and 6 older adults with uncontrolled hypertension, as well as 6 adults and 6 older adults with controlled BP. Finally, a total number of 24 participants were included in an in-depth interview.

#### Research Measurements

In phase 2, the researcher used a background information form, semistructured interviews, and an interview guide for data collection. The interview guide was comprised of a set of open-ended questions, including (1) How does hypertension affect your daily life? (2) After participating in the self-management activities combined with the web-based application, how do you feel your self-management behaviors have changed? (3) What are the factors that reinforce or hinder your self-care? (4) How has the web-based application helped you adjust your self-care behaviors? (5) What is the level of your satisfaction with the web-based application? (6) Which function(s) do you use most frequently? And why? and (7) What are the prominent features or any elements of the web-based application that you feel need improvement? The content validity was examined by 3 experts, including a nurse instructor specializing in self-management, a nurse instructor specializing in hypertensive care, and an instructor specializing in digital health. The content validity was 1.00.

#### Data Collection

In this phase, the study used in-depth and semistructured interviews and an interview guide to collect participants’ perceptions and experiences after completing all self-management activities combined with DHIs. The researcher conducted all interviews at a convenient time for the participants at the Siriraj Primary Care Unit for 30 minutes each in Thai, and each interview was audiorecorded.

#### Data Analysis

We will analyze the in-depth interviews in phase 2 using the method by Colaizzi [57]. The researcher will read the transcribed interviews multiple times to truly understand the feelings of the informants. After that, the researcher will thoroughly review each statement on every page to discern the narrative’s significance, understand the content’s significance and meaning, and define the meaning of the key phrases. The study will gather and thoroughly explain or describe the results obtained from the phenomenon under study. The study will clarify any ambiguous phenomena as much as possible and return the findings to the informants for verification.

### Ethical Considerations

The study was approved by the Human Research Ethics Committee of the Faculty of Nursing and the Human Research Ethics Committee of Siriraj Hospital Faculty of Medicine, which jointly considered and approved the research project in the form of a Memorandum of Understanding (MU-MOU COA number IRB-NS2025/948.3105). The study protocol was registered under a Universal Trial Number (U1111-1324-0264), and approved by the Thai Clinical Trials Registry (TCTR20250722001).

Additionally, we informed all participants about the study’s objectives, procedures, risks, and benefits. Participation was voluntary, and participants could withdraw at any time. Further, we required the participants to complete and sign consent forms before participation. Moreover, participants were able to stop attending the intervention at any time, before or after deciding to take part in this study, without any effects on their care at the Siriraj Primary Care Unit. In addition, the participants in the control group had access to the intervention upon request within 1 week after the intervention completion because this approach allowed us to minimize contamination and preserve the internal validity of the study while also ensuring that the participants in the control group could benefit from the intervention afterwards.

Further, the study keeps all collected data confidential following standard data protection laws (eg, participant anonymity was assured as the study would only disclose their participant numbers and initials) [58]. When study results are disseminated, only aggregated findings will be presented, ensuring that no individual participant can be identified. The researcher has stored the collected data in password-protected files on a personal computer, and documents related to the research participants have been kept in a locked cabinet accessible only to the researcher. Data stored on the cloud and audio recording files can only be accessed by the researcher with a password. After the research project is completed, the researcher will immediately destroy the data.

Moreover, the researcher covered each participant's travel expenses, totaling ฿160 (US $5.14) per trip. The researcher also covered the cost of a flat-rate internet service for each participant in the experimental group, at ฿300 (US $9.63) per month for 2 months, to support group activities and self-care practices at home. Each participant in the experimental group received compensation in 2 installments of ฿300 each, paid directly in weeks 1 and 4. In phase 2, the researcher paid each participant’s travel expenses for attending 1 in-depth interview, with a fee of ฿160 per interview. No additional compensation was provided for the interview.

## Results

This study received a grant in December 2024 and ethical approval on May 31, 2025. We recruited 86 individuals, including 18 adults and 68 older adults, with uncontrolled hypertension from the Siriraj Primary Care Unit in Thailand. In phase 1, this study began recruiting participants in August 2025. Data were collected between August 2025 and the first half of November 2025. In-depth interviews were conducted in phase 2 and continued through the end of November 2025. The data analysis should be completed by December 2025. The expected date for the results to be submitted for publication is March 2026.

Regarding the results presentation, the quantitative results from phase 1, including eating behavior, exercise behavior, stress management behavior, and BP, will be descriptively summarized by gender and age group. Age group and gender will be included as factors in the analyses, considering the limited sample size of the adult group. In phase 2, this study will use purposive sampling across age groups and BP status, and phase 2 will be conducted after completion of phase 1 to compare themes between groups and help explain how self-management combined with DHIs influences outcomes. Subsequent academic publications will highlight age-specific findings, including any identified digital challenges.

## Discussion

### Overview

This protocol describes the rationale and design of an explanatory sequential mixed methods study to evaluate the effectiveness of self-management combined with DHIs while also exploring participants’ experiences and perceptions after the intervention among patients with uncontrolled hypertension in Thailand. Uncontrolled hypertension is a significant public health issue in Thailand, leading to elevated morbidity, death, and increasing health care expenses. Previous evidence indicates that DHIs can support self-management and improve BP control [16,17], and their integration with nurses and other health care providers can further enhance adherence to self-management behaviors and BP control [21]. The researchers hypothesize that combining these self-management strategies with DHIs will improve dietary, exercise, and stress management behaviors and lower BP compared with standard care.

Existing research illustrated that DHIs have potential for improving both behavioral and clinical outcomes among patients with uncontrolled hypertension [16,17,22,30]. However, previous interventions in Thailand have emphasized single behaviors rather than adopting a comprehensive, multibehavior change approach [23,32,33 ]. The systematic review conducted by our team identified that the most effective DHIs for self-management among patients with uncontrolled hypertension address multiple health behaviors [21]. Our protocol addresses this gap by incorporating diet, exercise, and stress management strategies as core components that are often overlooked in interventions. In addition, several DHIs for patients with uncontrolled hypertension have been designed and focus on health education [22,30], medication reminders [22,30-33], and home BP monitoring [27,31,33,34] delivered through mobile apps, text messages, or web platforms. A key innovation of this study is a hybrid self-management model combined with DHIs targeting multiple behaviors.

The use of DHIs, such as web-based applications, chatbots, smartwatches, and salt meters, is not a one-size-fits-all solution. Several factors—including age group, digital health literacy, living area, and infrastructure—influence acceptance and uptake [59]. In this study, adults who are good with technology may find the web-based application and chatbot easy to use, whereas older adults who are digitally challenged may feel anxious or struggle with this technology, making it harder for them to use. To mitigate this, the researchers will incorporate a user-friendly interface with large text and initial in-person training sessions [60]. In addition, participants living in households with good infrastructure (good internet connectivity and affordability) may face fewer barriers than those living in households with poor infrastructure. To address this issue, the researchers will allocate a budget for internet connectivity and ease of internet access [61]. Recognizing these potential differences is important for correctly interpreting the study results.

An explanatory sequential design is used to address variations in users’ perceptions and experiences. The quantitative phase will estimate changes in dietary, exercise, and stress management behaviors, as well as BP, associated with the self-management combined with DHIs. The qualitative phase will provide a deeper understanding of why the intervention succeeds or fails in primary care units in Thailand. Researchers planned to purposefully sample both adults and older adults with either controlled or uncontrolled BP for in-depth interviews. This qualitative inquiry will uncover specific barriers to implementation, allowing the researchers to distinguish between intervention success or failure and identify barriers.

This protocol has two main strengths. First, this protocol is a theory-informed, multicomponent design that combines self-management approaches with DHIs to address multiple behaviors for uncontrolled hypertension. These interventions provide evidence-based information, develop self-management skills, and offer instrumental support, as outlined by the Individual and Family Self-Management Theory [51]. Finally, the use of an explanatory, sequential, mixed methods design allows us to examine both behavioral and clinical outcomes in depth, as well as the subtleties of user engagement. Nonetheless, limitations are anticipated. The requirement for participants to use their own smartphone devices, to have adequate digital health literacy, and to have data plans may create a selection bias, thereby omitting the most economically disadvantaged persons. Also, data synchronization may be interrupted in remote regions where the internet is not always available. Another limitation is that it is a single-center study conducted in a primary care unit in Thailand; therefore, generalizability may be limited. Because of ethical limits on collecting data from people who did not provide consent, the researchers could not compare participants with those who were excluded or chose not to take part. This means the findings may not generalize to people who are less comfortable with technology, underscoring how difficult it can be to close the digital divide in eHealth.

### Implications and Future Directions

The results of this study will have important effects on how Thailand deals with uncontrolled hypertension. If this combined self-management and DHI approach works, it could serve as a model for primary care units and noncommunicable disease clinics across the country. The explanatory sequence design is essential because it provides more than just data on effectiveness. It also helps us understand how factors like age, ability to use digital technology, and different experiences with health technologies influence outcomes.

Future research will focus on mitigating the gap of DHIs, including web-based applications and chatbots highlighted in this study. Insights from the qualitative phase on the digital divide and user acceptance will be critical for future DHI iterations. We aim to use these findings to develop more personalized, age-friendly interfaces or voice-activated features to further reduce barriers for patients with uncontrolled hypertension who are digitally challenged in Thailand. Furthermore, subsequent studies should evaluate the cost-effectiveness of this intervention to support policy decisions regarding its integration into the national health security system.

### Conclusion

Uncontrolled hypertension remains a critical public health challenge in Thailand, requiring innovative solutions that align with standard care. This protocol describes an innovative approach to addressing the knowledge gap between traditional self-management and DHIs, focusing on enhancing dietary, exercise, and stress management behaviors and reducing BP. This study aims to determine the effectiveness of self-management combined with DHIs and to explore participants’ experiences and perceptions after receiving the intervention. The results from this study will be used to determine the effectiveness of self-management combined with DHI solutions in primary care units in Thailand. Moreover, the results will have the potential to offer practical guidance on how to care for patients with uncontrolled hypertension, thereby improving their hypertensive outcomes. These results could also provide important information that will lead to the further development of health care policies in Thailand.

## Supplementary material

10.2196/81148 Checklist 1 SPIRIT Checklist.

## Abbreviations

BP: blood pressure
DASHQ: Dietary Approaches to Stop Hypertension Questionnaire
DHIs: digital health interventions
EBQ: Exercise Behavior Questionnaire
eHEALS: eHealth Literacy Scale
WHO: World Health Organization
